# Lead times in the early management of traumatic brain injury: relation to geographic conditions and clinical outcomes in a nationwide Swedish registry study

**DOI:** 10.1007/s00701-026-06817-3

**Published:** 2026-03-10

**Authors:** Amanda Gu, Francisco Leal-Méndez, Anders Lewén, Anders Hånell, Lina Holmberg, Per Enblad, Teodor Svedung-Wettervik, Fredrik Linder

**Affiliations:** 1 Department of Medical Sciences, Section of Neurosurgery, Uppsala University, Uppsala, Sweden; 2 Department of Surgical Sciences, Section of Vascular Surgery, Uppsala University, Uppsala, Sweden

**Keywords:** Lead time, Outcome, Swedish trauma registry, Traumatic brain injury, Trauma logistics

## Abstract

**Background:**

Traumatic brain injury (TBI) patients are at risk of sudden deterioration, requiring timely diagnostics and treatment to prevent secondary cerebral injuries. This study investigated lead times in prehospital and early intrahospital TBI management, assessing their association with geographical conditions, hospital caseloads, and patient outcomes.

**Methods:**

This nationwide, observational cohort study included 5036 TBI patients (during 2018–2022) from the Swedish Trauma Registry (SweTrau). Lead times from trauma to alarm, from alarm to hospital arrival, and times to first computed tomography (CT) from alarm and hospital arrival, respectively, were calculated. These were analyzed against the geographical distribution of healthcare, hospital caseloads, and 30-day mortality.

**Results:**

The majority of the cohort arrived in hospital within one hour and suffered a mild-to-moderate TBI. In univariate analyses, healthcare regions with larger geographical catchment areas exhibited longer time of prehospital management from alarm to arrival in hospital than smaller regions. Meanwhile, in multivariate linear regressions, larger region catchment area was independently associated with longer times from trauma to alarm and from alarm to hospital, but shorter time from alarm to first CT. In similar multivariate analyses, higher caseload was associated with longer time from alarm to first CT**.** Patients who were initially managed in a local hospital exhibited longer lead times overall, except from time to first CT from arrival in hospital. Furthermore, in the whole cohort, longer time from alarm to first CT and from arrival in hospital to first CT were associated with lower rate of mortality in univariate logistic regressions. However, this did not hold true in multivariate analysis after adjusting for demography and injury severity.

**Conclusions:**

Lead times in TBI management varied by both geographical and hospital-bound factors. Faster lead times in TBI were associated with higher mortality in univariate analysis, but this association disappeared in multivariate analysis, suggesting that clinical severity rather than time alone is the stronger predictor of outcome. Nonetheless, it remains believed that efficient and qualitative management is a fundamental necessity for better outcomes in TBI management.

## Introduction

Traumatic brain injury (TBI) affects approximately 70 million people annually and is a leading cause of mortality and morbidity worldwide [1, 8, 9]. Mild-to-moderate TBI patients typically present with symptoms such as headache, nausea, and amnesia. In some cases, there is a rapid clinical deterioration with loss of consciousness, unreactive pupils, and airway compromise, due to intracranial bleedings and elevated intracranial pressure (ICP) [8]. In addition, a subset of severe TBI cases present immediately in an unconscious state [7, 8, 8, 9]. Stable patients with mild TBI can typically be discharged from the emergency department or admitted for brief neurological monitoring without requiring further treatment [7, 8, 8, 8]. Patients with severe injuries or early deterioration are at high risk of mortality and need prompt physiological and neurosurgical management, primarily due to airway obstruction and ventilation failure caused by brain herniation following expanding intracranial hemorrhages [7, 7, 7, 9]. Additionally, patients with TBI are particularly vulnerable to secondary brain injuries resulting from hypoxia and hypotension, which may be aggravated by multi-trauma [8, 9].

Effective management of TBI requires immediate resuscitation, early diagnostics, and careful stratification to distinguish patients requiring emergent neurosurgery from those with mild injuries that can be managed conservatively. This necessitates efficient prehospital and in-hospital care pathways, which minimize delays from trauma to alarm activation, hospital arrival, imaging (trauma CT) and treatments. Prehospital care is particularly influenced by geographical variations in different hospital catchment areas, which may impact the time from injury to hospital arrival. Prehospitally, the need for immediate resuscitation must be weighed against the urgency of timely transport to a hospital capable of appropriate diagnostic imaging and advanced care [7, 7, 9]. The "Platinum Ten" principle recommends limiting on-site care to 10 min to avoid unnecessary delays while allowing sufficient time for initial stabilization [7, 8]. However, as shown in the multicenter CENTER-TBI study, prehospital practices vary considerably across Europe [9]. Subsequently, upon contact with the receiving hospital, a trauma alarm may be triggered to initiate immediate care, following local or national guidelines [8].

Evaluation and resuscitation of trauma patients follow the Advanced Trauma Life Support (ATLS) protocol prioritizing airway (A), breathing (B), circulation (C), neurological disability (D), and exposure (E) [7]. Hemodynamically unstable patients who do not respond to initial interventions may require immediate surgical interventions, while a trauma CT may be performed to evaluate potential injuries in stable patients [7, 8, 8, 9, 9]. For patients with isolated head trauma, protocols typically mandate that trained personnel assess the patient within 15 min of arrival [8]. TBI patients with mass lesions are often transferred to neurosurgical centers for hematoma evacuation and neurointensive care [7, 7, 7, 8, 8]. However, variations in hospital experience and caseload, and the distance to the nearest neurosurgical center can differ and influence the time from injury to definitive treatment [7, 9].

Sweden is a geographically large country with both densely populated urban areas and extensive sparsely populated regions, resulting in a substantial geographical variation in access to specialized neurosurgical care in terms of geographical distance and transportation time. The healthcare system is administratively divided into 20 individual health care regions, with a total of 49 local hospitals providing around the clock general trauma care, of which 7 university hospitals offer specialized neurosurgical care [1, 3, 7, 8]. The common practice is that TBI patients are stabilized initially at local hospitals, followed by secondary transfer to a neurosurgical center if needed [8, 8]. In regions maintaining a university hospital, patients may primarily be admitted to this specific neurosurgical department. Exceptionally, acute extracerebral hematomas may be evacuated in local hospitals as a life-saving procedure [8]. However, in geographically compact and densely populated regions, moderate to severe TBI patients are more often directly transported to hospitals with neurosurgical capabilities [8, 8]. Ultimately, it is evident that the geographical distribution of healthcare resources is uneven and may affect the management of TBI patients.

Many factors may influence the lapse of management of TBI patients, potentially delaying necessary diagnostics and treatments, and increasing risks of developing secondary brain injuries. There is limited evidence on how patient characteristics, geographical factors and hospital experience affect lead times in these early care pathways, and which impact delays have on clinical outcomes. Therefore, the aim of this study was to investigate variations in lead times, their explanatory variables and their effect on mortality in a Swedish nationwide cohort registry study.

## Materials and methods

### Study design and population

**Fig. 1:**
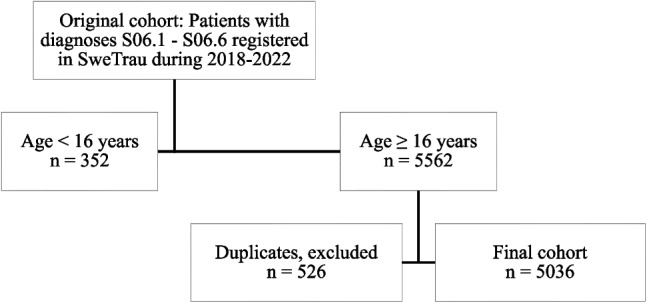
Flow chart of patient inclusion. In this study, individuals with diagnoses S06.1 - S06.6 aged ≥ 16 years were included and duplicate case registrations were excluded.

This retrospective observational study utilized data from the Swedish Trauma Registry (SweTrau), a nation-wide registry with data from hospitals in Sweden that provide care for severe traumatic injuries. The study focused on patients with TBI diagnoses and New Injury Severity Score > 15 (S06.1–S06.6), aged 16 or older, treated between January 1, 2018, and December 31, 2022. The data extracted with these inclusion criteria covered 20 Swedish health care regions and 47 hospitals. From the 5914 patients with these diagnoses during this time period, 352 were excluded due to age below 16. Furthermore, duplicate registrations were identified by person identification number or temporary identification number, age [years], date of birth, and gender. In total, 526 duplicate cases were excluded, resulting in a final cohort of 5036 individuals (Fig. 1).

### Data collection and variable definitions

All data used in this study were acquired from the SweTrau registry [7]. Demographics (age, sex), injury mechanisms (falls, road traffic accidents etc.), injury severity (Glasgow Coma scale [GCS], abbreviated injury scale [AIS] head, injury severity score [ISS]), injury types (epidural hematoma [EDH], acute subdural hematoma [ASDH], traumatic subarachnoid hemorrhage [tSAH], contusion), interventions (craniotomy) and outcome (30-day mortality) were extracted from the registry.

Registered date and time of trauma, alarm, primary hospital arrival and first CT, respectively, were also extracted. The accuracy of these time variables in SweTrau has previously been reported to be 74% or higher when allowing a margin of error up to 10 min, with a correctness of 89.7% of the registry data overall, and a case completeness of 100% for individuals with NISS > 15 [7]. The time intervals were calculated from trauma to alarm and from alarm to arrival in the primary hospital (local hospital or university hospital), which were considered indicators of access to care. Also, time to first CT from alarm and arrival in hospital was calculated, which were considered indicators of both pre- and in-hospital trauma management. Few individuals (*n* = 2) exhibited lead times of negative values which were treated as missing values. Outcome was dichotomized into mortality/survival 30 days post-injury.

### Statistical analysis

Statistical analyses were performed using SPSS (IBM SPSS Statistics, Version 29.0.2.0). Variables were described as medians (interquartile range (IQR)) or counts (proportions), depending on the data type. Differences in lead times for trauma management were analyzed in relation to geographical conditions (large vs. small counties), caseload (high vs. low), and clinical outcome (mortality vs. survival 30 days post-injury) using Mann–Whitney U-test. A multivariate linear regression was performed for each time interval (trauma to alarm, alarm to hospital, alarm to first CT, and hospital to first CT) as the dependent variable. The analyses were adjusted for hospital caseload (higher vs. lower), county size (larger vs. smaller), age, neurological injury severity (GCS) and type of initial hospital (university vs. local), to assess their independent associations. In addition, a multivariate logistic regression was performed with mortality as the dependent variable, to explore the independent association of the lead time variables after adjusting for demography (age and sex) and neurological injury severity (GCS). A *p*-value < 0.05 was considered statistically significant.

## Results

### Demography, management, lead times in management, injury severity, and outcome

As presented in Table 1, the median patient age was 65 years (IQR 46–78). The cohort was predominantly male (*n* = 3412, 68%), with a median pre-injury ASA score of 2 (IQR 1–3), and median GCS score at admission of 14 (IQR 12–15). The majority of patients were managed at university hospitals, either as primary or secondary cases (*n* = 3017, 59.9%).

**Table 1:** Demography, injury mechanisms, admission status, injuries, time logistics, treatments, and outcome

Variables			Entire cohort	
Patients, *n* (%)			5036 (100)	
Age (years), median (IQR)			65 (46–78)	
Sex (male/female), *n* (%)			Male 3412 (68)	
			Female 1624 (32)	
Pre-injury ASA score, median (IQR)			2 (1–3)	
Injury mechanism, *n* (%)		Traffic accident		1204 (23.9)
		Pedestrian accident		160 (3.2)
		Gunshot wound		21 (0.4)
		Penetrating trauma		20 (0.4)
		Blunt trauma		297 (5.9)
		Low energy fall		1850 (36.7)
		High energy fall		1268 (25.2)
		Injury from explosion		6 (0.1)
		Other (eg suffocation, burns)		101 (2.0)
		Missing		109 (2.0)
GCS at admission, median (IQR)		14 (12–15)		
GCS motor at admission, median (IQR)		6 (5–6)		
AIS head, median (IQR)		2 (1–3)		
Epidural hematoma, *n* (%) (S06.4)		533 (10.6)		
Acute subdural hematoma, *n* (%) (S06.5)		3758 (74.6)		
Traumatic subarachnoid hemorrhage, n (%) (S06.6)		2541 (50.5)		
Contusion, *n* (%) (S06.1)		191 (3.8)		
Managed at university hospital alone, local hospital alone, or both,* *n* (%)		University hospital alone		1733 (37.5)
		Local hospital alone		1608 (34.8)
		Both		1284 (27.8)
First admitting hospital, university hospital or local hospital,* *n* (%)		University hospital		2337 (46.4)
		Local hospital		2699 (53.6)
Trauma to alarm (minutes),* median (IQR)		29 (2–38)		
Alarm to hospital (minutes),* median (IQR)		45 (5–52)		
Alarm to first CT, * median (IQR)		153 (93–474)		
Hospital to first CT (minutes),* median (IQR)		70 (45–103)		
Craniotomy (yes), *n* (%)		587 (11.7)		
ICP-monitoring (yes), *n* (%)		481 (9.6)		
30-day mortality,* *n* (%)		930 (19)		
TBI severity*	Mild (GCS 13–15)	3035 (60.3)		
	Moderate (GCS 9–12)	540 (10.7)		
	Severe (GCS 3–8)	548 (10.9)		

*Missing data: Managed at university hospital alone, local hospital alone, or both: *n* = 413 (8.2%); First admitting hospital: *n* = 2 (0.0%); Trauma to alarm: *n* = 848 (16.8); Alarm to hospital: *n* = 847 (16.8); Alarm to first CT: *n* = 171 (3.4); Hospital to first CT: *n* = 170 (3.4); 30 day mortality: *n* = 113 (2.2%); TBI severity: *n* = 802 (15.9)%

Median lead times were as follows: injury to alarm 29 min (IQR 2–38), alarm to hospital arrival 45 min (IQR 5–52), and arrival to CT 70 min (IQR 45–103). The median AIS score for head injuries was 2 (IQR 1–3). In total, 587 (11.7%) underwent craniotomy, and 481 (9.6%) received ICP monitoring. The overall 30-day mortality rate was 19% (*n* = 930).

### Lead times of trauma management in relation to geographical factors and hospital caseload

The health care regions were dichotomized as smaller or larger based on the median geographical area per hospital (5713 km^2^), resulting in 10 smaller and 10 larger regions. As also shown in Table 2, geographically larger regions exhibited longer lead time between alarm to arrival in hospital (*p* < 0.05), but had no significant association to times from trauma to alarm, from alarm to first CT, or from hospital arrival to first CT. Hospitals were also dichotomized by caseload (Table 3), defined as low or high based on having a lower or higher annual volume of TBI patients than the median across all hospitals included in the registry data (46 cases in total, whereof 23 lower and 24 higher caseload hospitals, respectively). Hospital caseload was not significantly associated with any of the lead times.

**Table 2:** Time logistics of the trauma emergency care in relation to regional geography

Variables	Larger regions*		Smaller regions**		*p*-value
	*N* (%) valid	median (IQR)	*N* (%) valid	median (IQR)	
Trauma to alarm (minutes)	8 (80.0)	5 (3—8)	10 (100.0)	5 (5–5)	0.778
Alarm to hospital (minutes)	10 (100.0)	54 (52–64)	10 (100.0)	51 (48–51)	**0.041**
Alarm to first CT (minutes)	10 (100.0)	98 (95–120)	10 (100.0)	107 (95–107)	0.224
Hospital to first CT (minutes)	10 (100.0)	37 (34–47)	10 (100.0)	48 (39–48)	0.926

*Average catchment area per hospital within the same region > 5713 km^2^ (median of all regions)

**Average catchment area per hospital within the same region ≤ 5713 km^2^ (median of all regions)

**Table 3:** Time logistics of the trauma emergency care in relation to caseload

Variables	Higher caseload*		Lower caseload**		*p*-value
	*N* (%) valid	median (IQR)	*N* (%) valid	median (IQR)	
Trauma to alarm (minutes)	21 (91.3)	5 (2–45)	22 (91.7)	5 (1–81)	0.095
Alarm to hospital (minutes)	22 (95.7)	51 (38–69)	24 (100.0)	56 (39–79)	0.881
Alarm to first CT (minutes)	21 (91.3)	44 (28–108)	20 (83.3)	42 (30–68)	0.481
Hospital to first CT (minutes)	21 (91.3)	102 (75–149)	21 (87.5)	107 (81–144)	0.529

*Average case load per year > 67 (median of all hospitals)

**Average case load per year < 67 (median of all hospitals)

In a multivariate linear regression analysis of geographical area (smaller/larger) and caseload (low/high) in relation to the lead times of TBI management, adjustments were made for age, GCS score, and first admitting hospital (university/local hospital), as presented in Table 4. Higher hospital caseload correlated independently with longer time from alarm to CT (B = 34.71, *p* < 0.05) and from hospital arrival to CT (B = 43.99, *p* < 0.05), but showed no correlation to the other lead times. Larger region size was associated with overall longer lead times (*p* < 0.05), except for time from alarm to first CT which was shorter (B = −10.76, *p* < 0.05), and did not correlate to the time from hospital arrival to CT (*p* > 0.05).

**Table 4:** Multivariate linear regression analysis

Trauma to alarm			
	**Regression coefficient (B)**	**Standardized coefficients (β)**	***p*-value**
Caseload (lower/higher caseload)*	39.95	0.013	0.445
Size (smaller/larger regions)*	155.2	0.047	** < 0.001**
Age (years)	2.355	0.069	**0.003**
Gender (male/female)**	−29.88	−0.013	0.408
GCS in ED (scale)	0.099	0.020	0.216
Initial caregiver (university/local)**	61.18	0.038	**0.024**
Alarm to hospital			
	**Regression coefficient (B)**	**Standardized coefficients (β)**	***p*-value**
Caseload (lower/higher caseload)*	−0.271	−0.002	0.899
Size (smaller/larger regions)*	8.535	0.093	** < 0.001**
Age (years)	0.166	0.081	** < 0.001**
Gender (male/female)**	−1.678	−0.018	0.253
GCS in ED (scale)	0.013	0.065	** < 0.001**
Initial caregiver (university/local)**	2.935	0.045	**0.008**
Alarm to first CT			
	**Regression coefficient (B)**	**Standardized coefficients (β)**	***p*-value**
Caseload (lower/higher caseload)*	34.71	0.108	** < 0.001**
Size (smaller/larger regions)*	−10.76	−0.047	**0.002**
Age (years)	0.661	0.129	** < 0.001**
Gender (male/female)**	1.936	0.008	0.569
GCS in ED (scale)	−0.008	−0.016	0.297
Initial caregiver (university/local)**	26.33	0.160	** < 0.001**
Hospital to first CT			
	**Regression coefficient (B)**	**Standardized coefficients (β)**	***p*-value**
Caseload (lower/higher caseload)*	43.99	0.043	**0.006**
Size (smaller/larger regions)*	−11.21	−0.015	0.294
Age (years)	0.213	0.013	0.382
Gender (male/female)**	−3.508	−0.005	0.751
GCS in ED (scale)	0.009	0.005	0.725
Initial caregiver (university/local)**	−36.85	−0.070	** < 0.001**

*Regression coefficients are presented for higher hospital caseload, larger geographical region size

**Categorical variables, regression coefficients are presented for female gender and local hospital

Higher age was associated with longer time from trauma to alarm, from alarm to hospital and to CT, but not from hospital to CT (*p* > 0.050). Patients with lower GCS showed faster time from alarm to hospital, meanwhile, gender was not associated with any of the lead times (*p* > 0.05). Having been initially managed in a local hospital was associated with longer time from trauma to alarm, from alarm to hospital arrival, and from alarm to CT (all *p* < 0.05), but shorter time from hospital to CT (*p* < 0.05).

Lead times of trauma management in relation to patient outcomes.

In a univariate analysis, patients who survived presented with significantly longer time from alarm to CT, and hospital to CT, but shorter time from trauma to alarm, as seen in Table 5. Meanwhile, time from alarm to hospital was not associated with higher mortality (p > 0.05) (Table 5).

**Table 5:** Time logistics of the trauma emergency care in relation to survival and mortality

Variables	Survivors		Deceased		*p*-value
	*N* (%) valid	median (IQR)	*N* (%) valid	median (IQR)	
Trauma to alarm (minutes)	3264 (81.7)	5 (2–27)	846 (91.2)	11 (3–380)	** < 0.001**
Alarm to hospital (minutes)	3264 (81.7)	52 (38–70)	846 (91.2)	52 (37–67)	0.096
Alarm to first CT (minutes)	3472 (81.3)	107 (77–158)	660 (86.3)	87 (71–119)	** < 0.001**
Hospital to first CT (minutes)	3265 (81.7)	48 (29–115)	868 (93.5)	37 (26–58)	** < 0.001**

In a multivariate logistic regression analysis, seen in Table 6, longer time from trauma to alarm was associated with higher mortality (OR 1.000, 95% CI (1.000–1.000), *p* < 0.05). Furthermore, there was no other independent association between any of the lead times and mortality, after adjustment for age, gender, GCS score, head AIS score, and pre-injury ASA score. Higher risk of mortality was associated with increasing age (OR 1.052, 95% CI (1.046–1.058), *p* < 0.05) and with higher pre-injury ASA score (OR 1.001, 95% CI (1.000–1.002), *p* < 0.05). Meanwhile, lower AIS head score showed independent association with lower mortality (OR 0.934, 95% CI (0.891–0.979), *p* < 0.05). Neither GCS nor gender was significantly associated with mortality.

**Table 6:** Multivariate logistic regression analysis

Variables	Mortality	
	OR (95% CI)	*p*-value
Age (years)	1.052 (1.046–1.058)	** < 0.001**
ASA pre-injury (score)	1.001 (1.000–1.002)	**0.033**
AIS head (score)	0.934 (0.891–0.979)	**0.005**
Gender (male vs female) *	0.844 (0.706–1.009)	0.063
GCS (sum)	1.000 (1.000–1.001)	0.085
Trauma to alarm (minutes)	1.000 (1.000–1.000)	** < 0.001**
Alarm to hospital (minutes)	1.042 (0.896–1.213)	1.042
Alarm to first CT (minutes)	0.957 (0.822–1.113)	0.568
Hospital to first CT (minutes)	1.037 (0.891–1.207)	0.638

*Categorical variable, OR and 95% CI is presented for female gender

## Discussion

In this large nationwide study of 5036 TBI patients, the main findings were that most patients arrived at a hospital within one hour of injury, while geographically larger regions exhibited longer prehospital management. More severe neurological and systemic injuries, indicated by lower GCS, were generally associated with shorter time to first CT. Univariate analyses showed overall longer in-hospital management among survivors, although, these relationships did not persist in multivariate regressions*.* However, the lead times also exhibited a complex interplay with other factors including injury severity, geographical conditions, and resource availability.

Our findings showed that Swedish prehospital management was generally effective, with most cases arriving at a hospital within an hour. However, there was substantial variation in pre- and in-hospital lead times. Firstly, there were several important factors related to the healthcare organization. Larger geographical county area was independently associated with longer prehospital lead times, consistent with greater transportation distances, but shorter time from arrival in hospital to first CT. The prehospital lead times were also longer at local hospitals, compared to university hospitals, while the opposite was true for in-hospital lead time from hospital arrival to first CT. One possible explanation is that university hospitals are often located in Sweden’s larger cities, where the proximity between patient and hospital and the density of prehospital resources, including helicopter emergency medical services, are generally higher. However, a potential drawback is the typically high patient load at these emergency departments, leading to increased competition for rapid assessment and imaging resources with other critically ill patients. In contrast, a major trauma case at a smaller hospital is more uncommon and may be prioritized more rapidly throughout the chain of care, leading to shorter in-hospital lead times. Consistent with this idea, higher caseload hospitals exhibited slower in-hospital lead times. While a high volume of cases could theoretically lead to greater efficiency through routine and experience [7, 7, 7–9, 9], this potential advantage was likely outweighed by the strain on resources and bottlenecks associated with managing many critically ill patients simultaneously [7, 8].

Secondly, there were also several important patient-specific factors related to the lead times in trauma management, and to mortality. Older patients consistently exhibited longer lead times. Moreover, the presence of comorbidities makes early management more complex, and older or more frail patients may require more thorough assessment and stabilization before proceeding to imaging or definitive care. Also, offering the full extent of advanced trauma care may not always be appropriate or beneficial in this patient group, and individualized decisions regarding the level of intervention are often required. As expected, patients with more severe neurological injuries exhibited shorter prehospital lead times, probably as they received high-priority to receive necessary diagnostics and possibly in-hospital emergency neurosurgery [8]. This may have contributed to the increased risk of mortality associated with shorter prehospital leadtimes, observed in univariate analysis. Furthermore, a study of more severe injuries in an older cohort by Region Stockholm showed early transfers to be associated with poorer outcomes [7]. This supports the hypothesis that patients in greatest need of acute care are likely identified and transferred promptly, even though such cases often represent a particularly vulnerable and clinically complex subgroup.

Regarding the clinical significance of lead times on outcome, univariate analyses in this study showed that patients who survived exhibited longer lead times to CT and definitive care. This was likely confounded by injury severity, as survivors tended to have milder injuries with less urgent need for intervention. Consistently, in multivariate analysis, adjusting for such clinical variables, no independent association between lead times and outcome could be demonstrated. This suggests that, at the group level, time intervals in trauma management may be of lower prognostic relevance compared to established predictors such as age and neurological injury severity as measured by GCS, and AIS head score, as major predictors of mortality in TBI [7, 7–9]. However, higher pre-injury ASA score also showed to associate independently with higher mortality, in agreement with past research [8, 9], enhancing the significance of patient-bound conditions in the outcome after TBI.

Although the concept that "time is brain" remains highly relevant in case of impending brain herniation, this represents a relatively uncommon and dynamic subset in the entire spectrum of TBI eliciting a trauma alarm, as opposed to selected severe cases admitted to neurointensive care units [9]. In the current cohort, despite triggering trauma team activation due to suspected severe trauma, many patients presented with GCS scores within the mild-to-moderate range. Moreover, even in severe TBI, the number of patients requiring immediate neurosurgery with evacuation of intracranial bleedings may be limited, as a substantial proportion is unconscious due to factors not related to mass effect such as traumatic axonal lesions. Another aspect related to early diagnostics is the risk of intracranial bleeding progression, particularly among patients on anticoagulant therapy [7]. While such data were not available in this study, due to not being presented in the SweTrau registry, previous research has shown a clear benefit of early reversal of warfarin [9] and potential effects of prothrombin complex concentrates and tranexamic acid in patients on novel oral anticoagulants [9]. Nevertheless, these nuances may not shift overall outcome patterns at the group level, not least because trauma systems continuously strive to compensate for their weakest links. Clinical deterioration is often met with prompt countermeasures, and adverse events may be mitigated by e.g., emergency neurosurgery before causing lasting harm. Additionally, high-quality trauma care encompasses more than just rapid access to emergency neurosurgery. Timely resuscitation, with attention to airway, breathing, and circulation (ABCDE), is essential to avoid secondary brain injury from hypoxia and hypotension, both of which are well-established predictors of poor outcome in TBI [9, 9]. Still, the optimal timing for intervention is not always in the emergency room: extended prehospital time may, in some cases, be justified by the need for airway protection or hemodynamic stabilization, potentially mitigating the harm of secondary insults before hospital arrival [9].

Ultimately, while specific patient subgroups such as those with herniation syndromes or anticoagulated patients with intracranial hemorrhage may benefit from faster intervention, the complex interplay between injury severity, physiological response, and care quality makes it difficult to isolate time as a primary driver of outcome. In this cohort, characterized by a predominance of mild-to-moderate TBI, the observed lack of association between time and outcome reinforces the notion that age and clinical severity, rather than lead times per se, remain the most robust predictor of prognosis in TBI.

### Methodological considerations

The study has many strengths. It is based on a large national cohort of more than 5000 TBI patients with comprehensive data coverage. Missing data was relatively rare, although certain variables had lower data availability.

The extracted times of events from the registry data, used to calculate lead time intervals, may have been imprecise (± 1 h), due to registration in SweTrau being performed retrospectively. This introduces uncertainty, particularly regarding the time intervals which were less than one hour. Although this uncertainty may have contributed to incomprehensive results, as mentioned, the used variables have been shown to have a correctness of 74% or higher for this patient group when allowing a margin of error up to 10 min [7]. Moreover, previous studies on TBI [7, 7] have concluded that exact prehospital and in-hospital timings are not the primary determinants of patient outcomes, foreshadowing doubt to the significance of this uncertainty.

In this study, having a regional hospital as the first admitting hospital was associated with longer time from trauma to alarm, alarm to hospital arrival, and alarm to CT, but shorter time from trauma and from arrival to CT. This may be caused by a higher proportion of missing data for time from trauma to alarm and alarm to hospital arrival, compared to the lead times which were independent of the time of alarm. However, this higher rate of missing data may also be caused by registration of patients who suffered TBI while already inpatient.

While including a predominant share of mild TBI, and with a median cohort ASA score of 2, the mortality rate in this cohort was 18.4%. As the cause of death is not registered in SweTrau, it is possible that a proportion of these deaths were attributed to comorbidities or systemic injuries injuries. Also, given regards that the mortality was measured as of 30 days after injury, it cannot be disregarded that the patient deceased from a separate insult not related to TBI. Furthermore, the registry does not include information about the cause of death. Thus, especially in patients suffering multiple traumatic injuries, it cannot be certain that the patient deceased as a direct cause of the TBI. However, the overall mortality of all registered cases in SweTrau during the period of 2022–2024 was approximately 16% [2], and injury to the central nervous system and multi-organ failure are leading causes of death in patients suffering polytrauma [8, 9]. Nevertheless, the lack of this data is considered a limitation in evaluating patient outcome. Furthermore, the SweTrau registry does not include information on the prevalence of withholding of treatment or choosing not to escalate treatment. With regards to the median age of 65 years in the cohort of this study, accountability for such circumstances should be taken when observing the outcomes in TBI.

## Conclusions

Many patients in this nationwide cohort arrived at a hospital within one hour of injury and underwent a first trauma CT within two hours. Geographically larger regions exhibited longer prehospital management, while university hospitals and higher caseload hospitals showed longer overall lead times but shorter in-hospital time to first CT. Severe neurological injuries were generally associated with more rapid trauma management, reflecting higher prioritization. Univariate analyses indicated that faster lead times were associated with higher mortality, while no such association was found in multivariate regressions. These findings suggest that lead times in TBI management interact intricately with both care-related and external factors, including injury severity, geographical challenges, system organization, and resource availability. While timely care, particularly in subgroups requiring urgent neurosurgical intervention or hemodynamic stabilization remains crucial, this study reinforces the view that clinical severity markers such as age and GCS are stronger predictors of outcome than the lead times in TBI management.

## Data Availability

The data is available upon reasonable request.

## Abbreviations

AIS: Abbreviated Injury Scale
ASA: American Society of Anesthesiologists
ASDH: Acute Subdural Hematoma
ATLS: Advanced Trauma Life Support
BP: Blood pressure
CI: Confidence interval
CPR: Cardiopulmonary resuscitation
CT: Computed tomography
EDH: Epidural hematoma
EMS: Emergency medical services
GCS: Glasgow Coma Scale
HEMS: Helicopter emergency medical services
ICP: Intracranial pressure
ISS: Injury Severity Score
IQR: Interquartile range
NISS: New Injury Severity Score
OR: Odds-ratios
PaCO_2_: Partial pressure of CO_2_
sBP: Systolic blood pressure
SpO_2_: Peripheral oxygen saturation
SweTrau: Swedish Trauma Registry
TBI: Traumatic brain injury
Tsah: Traumatic subarachnoidal hemorrhage
WSES: World Society of Emergency Surgery
